# The complete genome sequence of *Ensifer meliloti* strain CCMM B554 (FSM-MA), a highly effective nitrogen-fixing microsymbiont of *Medicago truncatula* Gaertn

**DOI:** 10.1186/s40793-017-0298-3

**Published:** 2017-12-13

**Authors:** Marianna Nagymihály, Bálint M. Vásarhelyi, Quentin Barrière, Teik-Min Chong, Balázs Bálint, Péter Bihari, Kar-Wai Hong, Balázs Horváth, Jamal Ibijbijen, Mohammed Amar, Attila Farkas, Éva Kondorosi, Kok-Gan Chan, Véronique Gruber, Pascal Ratet, Peter Mergaert, Attila Kereszt

**Affiliations:** 1 Biological Research Centre, Hungarian Academy of Sciences, Szeged, Hungary; 2 Institute for Integrative Biology of the Cell, UMR 9198, CNRS/Universite Paris-Sud/CEA, 91198 Gif-sur-Yvette, France; 3 Seqomics Biotechnology Ltd, Mórahalom, Hungary; 4 Division of Genetics and Molecular Biology, Institute of Biological Sciences, Faculty of Science, University of Malaya, Kuala Lumpur, Malaysia; 5 UM Omics Centre, University of Malaya, Kuala Lumpur, Malaysia; 6 Laboratory of Soil Microbiology and Environment, Université Moulay Ismail, Meknes, Morocco; 7 Moroccan Coordinated Collections of Micro-organisms, Laboratory of Microbiology and Molecular Biology, National Center for Scientific Research, Rabat, Morocco; 8 Institute of Plant Sciences Paris Saclay IPS2, 91198 Gif-sur-Yvette, France

**Keywords:** *Ensifer meliloti*, Root nodule bacteria, Nitrogen-fixation, Symbiosis

## Abstract

Strain CCMM B554, also known as FSM-MA, is a soil dwelling and nodule forming, nitrogen-fixing bacterium isolated from the nodules of the legume *Medicago arborea* L. in the Maamora Forest, Morocco. The strain forms effective nitrogen fixing nodules on species of the *Medicago*, *Melilotus* and *Trigonella* genera and is exceptional because it is a highly effective symbiotic partner of the two most widely used accessions, A17 and R108, of the model legume *Medicago truncatula* Gaertn. Based on 16S rRNA gene sequence, multilocus sequence and average nucleotide identity analyses, FSM-MA is identified as a new *Ensifer meliloti* strain. The genome is 6,70 Mbp and is comprised of the chromosome (3,64 Mbp) harboring 3574 predicted genes and two megaplasmids, pSymA (1,42 Mbp) and pSymB (1,64 Mbp) with respectively 1481 and 1595 predicted genes. The average GC content of the genome is 61.93%. The FSM-MA genome structure is highly similar and co-linear to other *E. meliloti* strains in the chromosome and the pSymB megaplasmid while, in contrast, it shows high variability in the pSymA plasmid. The large number of strain-specific sequences in pSymA as well as strain-specific genes on pSymB involved in the biosynthesis of the lipopolysaccharide and capsular polysaccharide surface polysaccharides may encode novel symbiotic functions explaining the high symbiotic performance of FSM-MA.

**Electronic supplementary material:**

The online version of this article (doi: 10.1186/s40793-017-0298-3) contains supplementary material, which is available to authorized users.

## Introduction

To secure their nitrogen supply, legumes such as alfalfa, pea, (soy−/faba-)bean establish an endosymbiotic interaction with soil bacteria collectively called rhizobia that can reduce atmospheric nitrogen gas and produce reduced nitrogen molecules metabolizable by the plants. This symbiosis between legumes and rhizobia is of ecological and economic importance because of its contribution to the global nitrogen cycle, its impact on sustainable agriculture and its biotechnological potential to ensure nitrogen supply in agriculture [1].

The reduction of atmospheric nitrogen by rhizobia takes place in a specific niche, within the cells of *de novo* formed organs called nodules found usually on the roots and in some cases on the stem of the plants. Nodule development is initiated when flavonoids released by the plants induce the expression of the bacterial nodulation (*nod*) genes resulting in the production of the lipo-chitooligosaccharide signal molecules, the Nod factors. Nod factors cause a change in the direction of polar growth in developing root hairs and simultaneously induce cell division in the root cortex cells. As a result, a nodule primordium is formed that turns into meristematic tissue to produce the cells of the nodule and bacteria become entrapped in the curled root hair where they form an infection pocket. From the site of the infection pocket, a tubular structure, called infection thread, is formed in the root hair that grows toward the cells of the developing nodule. In the infection thread, bacteria multiply and finally they are released into the cytoplasm of the nodule cells via a mechanism resembling endocytosis resulting in organelle-like structures called symbiosomes. Symbiosomes have a membrane of plant origin which surrounds one or more bacteria. After bacterial release, the cells of both partners differentiate into mature symbiotic cells. The nodule cells become enlarged polyploid cells which host several tens of thousands of bacteria that are themselves differentiated into a nitrogen-fixing form called bacteroid [2–4]. Interestingly, in *Medicago* and closely related species like *Pisum* and *Vicia*, the host imposes a terminal differentiation on the bacterial partner that is accompanied by the increase in the DNA content and size of the bacteroids and results in the loss of their cell division capacity [5]. This terminal differentiation is orchestrated by nodule-specific cysteine-rich peptides that are expressed exclusively in the infected cells of the nodule [6, 7].

To effectively investigate these interactions, two genetic model legume species, *Lotus japonicus* (Regel) K. Larsen (bird’s-foot trefoil) and *Medicago truncatula* Gaertn. (barrel clover/barrel medic) have been chosen for which structural and functional genomics tools and databases have been developed [8, 9]. *M. truncatula* is a diploid, self-pollinating annual plant belonging to the *Medicago* genus, which contains species that are among the most extensively cultivated forage and pasture plants. *Medicago* plants establish symbiosis only with a limited number of bacterial species, mainly with 10.1601/nm.1328 (synonym 10.1601/nm.1339) *meliloti* and 10.1601/nm.1334, and with certain 10.1601/nm.1331 strains and 10.1601/nm.1298 [10–12]. However, some combinations of wild-type plants (species, sub-species and ecotypes) and bacterial strains of the most-studied bacterial species, 10.1601/nm.1335 and 10.1601/nm.1334, often lead to incompatible interactions [13–17], i.e. nodule formation is initiated but bacteria cannot invade nodules or cannot persist and fix nitrogen in the symbiotic organ. The incompatibility can be caused by functions/proteins encoded by genes in the accessory genome of the bacteria [14] such as the strain-specific HrrP peptidase [18], strain specific exopolysaccharide production [19] and/or allelic variants of the host genes like the *NFS1* and *NFS2* genes encoding NCR peptides in *M. truncatula* [20, 21]. Strikingly, the model bacterium 10.1601/nm.1335 strain 1021 (with the reference genome and most of the available mutants) is poorly matched for nitrogen fixation with the most widely used *M. truncatula* accessions Jemalong A17 and *M. truncatula* ssp. *tricycla* R108 [22, 23].

10.1601/nm.1335 strain FSM-MA (first catalogued as 10.1601/nm.1330 *strain* 10.1601/strainfinder?urlappend=%3Fid%3DCCMM+B554, also known as LMG-R33403 and MR372) was isolated from the nodules of *Medicago arborea* L. (moontrefoil/tree medic) in Maamora Forest between Rabat and Meknes, Morocco, and is stored in The Moroccan Coordinated Collections of Microorganisms as 10.1601/strainfinder?urlappend=%3Fid%3DCCMM+B554. Recently, Kazmierczak et al. [22] identified 10.1601/nm.1335 strain FSM-MA as a highly effective symbiotic partner of the two most widely used *M. truncatula* ecotypes, A17 and R108, as well as all tested *Medicago sativa* L. (alfalfa) cultivars. To gain the potential to identify novel bacterial symbiotic genes and genes associated with FSM-MA’s exceptional symbiotic performance, we sequenced the genome of the strain FSM-MA. Here we present a summary classification and a set of general features for 10.1601/nm.1335 strain FSM-MA, together with a description of its genome sequence and annotation.

## Organism information

### Classification and features

**Fig. 1:**
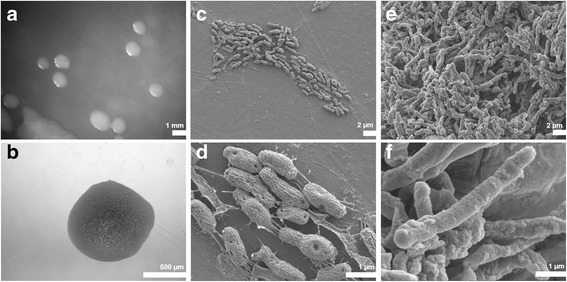
Colony morphology of *E. meliloti* strain FSM-MA on solid medium (**a**,**b**) at 5× (*A*) and 50× (**b**) magnifications as well as scanning electron microscopic images at 4000× (**c,e**) and 20,000× magnifications (**d,f**) of free-living cells (**c,d**) and bacteroids isolated from *M. truncatula* Jemalong A17 nodules (**e**,**f**)

10.1601/nm.1335 FSM-MA is a motile, non-sporulating, Gram-negative strain (Fig. 1) in the order 10.1601/nm.1277 of the class 10.1601/nm.809. This fast growing strain forms colonies within 3 days on YEB agar plates [22] at 30 °C. The colonies (Fig. 1a, b) are light beige colored on YEB plates, slightly doomed, mucoid and have a smooth margin. The rod shaped free-living form (Fig. 1c, d) has dimensions of 1.0–2.0 μm in length and approximately 0.5 μm in width, while bacteroids in *M. truncatula* Jemalong A17 nodules (Fig. 1e, f) have the same width and are elongated to 5–8 μm. A summary of the classification is provided in Table 1.

**Table 1:** Classification and general features of E. meliloti strain FSM-MA

MIGS ID	Property	Term	Evidence code^a^
	Current classification	Domain *Bacteria*	TAS [32]
		Phylum *Proteobacteria*	TAS [33]
		Class *Alphaproteobacteria*	TAS [34, 35]
		Order *Rhizobiales*	TAS [35–37]
		Family *Rhizobiaceae*	TAS [37, 38]
		Genus *Ensifer*	TAS [39–43]
		Species *Ensifer meliloti*	TAS [40, 42]
		Strain FSM-MA (B554)	
	Gram stain	Negative	IDA
	Cell shape	Rod	IDA
	Motility	Motile	IDA
	Sporulation	Non-sporulating	NAS
	Temperature range	Mesophile	NAS
	Optimum temperature	28–37 °C	IDA
	pH range	5.5–9.5	IDA
	Carbon source	Various	TAS [44]
GS-6	Habitat	Soil, root nodule on hosts	IDA
MIGS-6.3	Salinity	Unknown	NAS
MIGS-22	Oxygen requirement	Aerobic	NAS
MIGS-15	Biotic relationship	Free living, Symbiotic	IDA
MIGS-14	Pathogenicity	Non-pathogen	TAS [45]
	Energy source	Chemoorganotroph	NAS
MIGS-14	Pathogenicity	Non-pathogenic	NAS
MIGS-4	Geographic location	Maamora Forest, Morocco	NAS
MIGS-5	Sample collection	2004	NAS
MIGS-4.1	Latitude	Not reported	NAS
MIGS-4.2	Longitude	Not reported	NAS
MIGS-4.4	Altitude	Not reported	NAS

^a^Evidence codes - IDA: Inferred from Direct Assay; TAS: Traceable Author Statement (i.e., a direct report exists in the literature); NAS: Non-traceable Author Statement (i.e., not directly observed for the living, isolated sample, but based on a generally accepted property for the species, or anecdotal evidence). These evidence codes are from the Gene Ontology project [46] (http://geneontology.org/page/guide-go-evidence-codes)

#### Extended feature descriptions

**Fig. 2:**
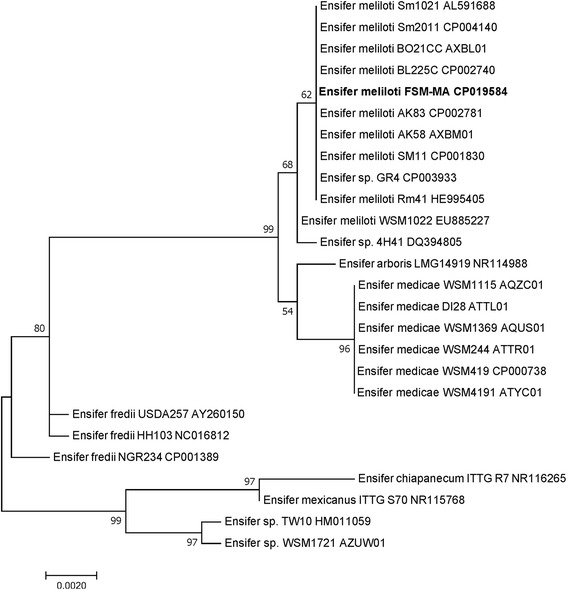
Phylogenetic tree showing the relationship of *E. meliloti* strain FSM-MA. The evolutionary history was inferred by using the Maximum Likelihood method based on the General Time Reversible model [30]. The tree with the highest log likelihood (−2208.71) is shown. The percentage of trees in which the associated taxa clustered together is shown next to the branches. Initial tree(s) for the heuristic search were obtained automatically by applying Neighbor-Join and BioNJ algorithms to a matrix of pairwise distances estimated using the Maximum Composite Likelihood (MCL) approach, and then selecting the topology with superior log likelihood value. The tree is drawn to scale, with branch lengths measured in the number of substitutions per site. Evolutionary analyses were conducted in MEGA7 [31]

Phylogenetic analysis of 10.1601/nm.1335 strain FSM-MA was performed by aligning the 16S rRNA sequence to the 16S rRNA sequences (consensus sequence length of 1346 basepairs (bp)) of other 10.1601/nm.1328 strains (Fig. 2). The FSM-MA 16S rRNA sequence has 100% sequence identity with those of the widely used 10.1601/nm.1335 strains such as strain 1021 or Rm41, while four mismatches can be observed with the 10.1601/nm.1334 strain 10.1601/strainfinder?urlappend=%3Fid%3DWSM+419 sequence. Moreover, there are five mismatches between the 16S rRNA sequence fragments of strain FSM-MA and 10.1601/nm.1330 strain 10.1601/strainfinder?urlappend=%3Fid%3DLMG+14919 ^T^. A Multilocus Sequence Analysis (Additional file 1: Figure S1) using 14 chromosomal genes further confirmed FSM-MA as an 10.1601/nm.1335 strain and clearly separated it from 10.1601/nm.1330 strain 10.1601/strainfinder?urlappend=%3Fid%3DLMG+14919 ^T^, 10.1601/nm.1334 strain 10.1601/strainfinder?urlappend=%3Fid%3DWSM+419 and the 10.1601/nm.1331 strains 10.1601/strainfinder?urlappend=%3Fid%3DNGR+234, 10.1601/strainfinder?urlappend=%3Fid%3DUSDA+257 and HH103. Among the 10.1601/nm.1335 strains, strain FSM-MA is most closely related to strains BO21CC and BL225C which were isolated from *M. sativa* nodules in Lodi, Italy [24]. Finally, the two-way average nucleotide identity [25] was calculated between genomes using the default settings of the ANI calculator. The genome of strain FSM-MA showed 99,42% identity with the genome of 10.1601/nm.1335 strain 1021 and only 90,09% identity with the genome of 10.1601/nm.1330 strain 10.1601/strainfinder?urlappend=%3Fid%3DLMG+14919 ^T^ *,* 87,09% identity with the genome of 10.1601/nm.1334 strain 10.1601/strainfinder?urlappend=%3Fid%3DWSM+419 and 83,16% and 83,31% identity with the genomes of 10.1601/nm.1331 strains 10.1601/strainfinder?urlappend=%3Fid%3DNGR+234 and HH103, respectively. Once more this analysis showed that FSM-MA is an 10.1601/nm.1335 strain and not an 10.1601/nm.1330 strain, considering a cut-off for species delineation at 95% identity [25].

#### Symbiotaxonomy

Strain FSM-MA forms effective nitrogen fixing nodules on *Medicago* species *M. sativa* L., *M. truncatula*, *M. arborea* L., *M. sativa subsp. x varia*, *M. ruthenica* (L.) Trautv. as well as on *Trigonella calliceras* Fisch., *Melilotus albus* (L.) Lam. (white sweetclover) and *Melilotus officinalis* (L.) Lam. (yellow sweetclover). Moreover, in agreement with its classification as 10.1601/nm.1335, it nodulates *Medicago polymorpha* L. (burclover) –that forms nitrogen-fixing symbiosis with 10.1601/nm.1334 strains – but there is no nitrogen fixation in the formed nodules.

## Genome sequencing information

### Genome project history

This organism was selected for sequencing on the basis of its superior symbiotic performance [22] with the most widely used accessions (A17 and R108) of the model legume *M. truncatula*. The genome project and the sequence of the three replicons are deposited in the National Center for Biotechnology Information (NCBI; accession numbers: CP019584, CP019585, CP019586). Genome sequencing and sequence assembling were performed at the University of Malaya (Kuala Lumpur, Malaysia) and at the Seqomics Biotechnology Ltd. (Mórahalom, Hungary). Annotation was carried out at Seqomics Biotechnology Ltd. A summary of the project information can be found in Table 2.

**Table 2:** Genome sequencing project information for *E. meliloti* strain FSM-MA

MIGS ID	Property	Term
MIGS-31	Finishing quality	Finished
MIGS-28	Libraries used	Illumina mate-paired library
		PacBio SMRTbell library
MIGS-29	Sequencing platforms	Illumina MiSeq
		PacBio RS II
MIGS-31.2	Fold coverage	249.2×
MIGS-30	Assemblers	CLC Genomic Worknbench v. 9.5; HGAP v. 3
MIGS-32	Gene calling methods	Genemark S+, used as part of the NCBI Prokaryotic Genome Annotation Pipeline PGAP
	Locus Tag	SMB554
	Genbank ID	CP019584-CP019586
	Genbank Date of Release	2017.07.01
	GOLD ID	Gp0258805
	BIOPROJECT	PRJNA369312
MIGS-13	Source Material Identifier	FSM-MA
	Project relevance	Symbiotic Nitrogen-fixation, agriculture

### Growth condition and genomic DNA preparation

10.1601/nm.1335 strain FSM-MA was grown on solid YEB medium (0.5% beef extract; 0.1% yeast extract; 0.5%peptone; 0.5% sucrose; 0.04% MgSO_4_.7H_2_O; pH 7.5) for 3 days and a single colony was used to inoculate 3 ml YEB broth medium. The culture was grown for 24 h on a gyratory shaker at 225 rpm at 30 °C, then 0.5 ml of the starter culture was used to inoculate 50 ml YEB broth medium. The culture was grown at 30 °C at 225 rpm until OD_600_ = 0.6 was reached. DNA was isolated from the cells with the MasterPure Complete DNA and RNA Purification Kit (Epicentre). The integrity of the extracted genomic DNA was analyzed by 0.7% agarose gel electrophoresis. The final concentration of the DNA, estimated with the help of a Qubit Fluorometer (ThermoFisher Scientific), was 0.45 mg ml^−1^.

### Genome sequencing and assembly

The genome sequence of 10.1601/nm.1335 strain FSM-MA was generated using Pacific BioScience (PacBio) and Illumina technologies. An Illumina Mate Paired library (average insert length 7 kbp) was constructed and sequenced using the Illumina MiSeq platform, which generated 3,387,162 reads. Similarly, a PacBio SMRTbell library was constructed and sequenced on the PacBio RS II platform to generate 254,443 filtered reads (N50 value at 8643 bp and total bases at 1,726,776,880 bp). Assembly was then carried out using HGAP version 3 [26] yielding three contigs with an average coverage of 186.71×. Subsequently, Illumina reads were aligned to the PacBio assembly with the help of the CLC Genomics Workbench version 9.5 and the observed 17 InDels were corrected. The final assembly contains three circular contigs corresponding to the three replicons (the chromosome and the pSymA and pSymB megaplasmids) totaling 6,703,999 bp and total input read coverage was at 249.2×.

### Genome annotation

Genes were identified and annotated using the NCBI Prokaryotic Genome Annotation Pipeline. The NCBI non-redundant database, UniProt, TIGR/Fam, Pfam, PRIAM, KEGG, COG, and InterPro databases were used to analyse the predicted coding sequences after translation. HMMER [27] and tRNAscan-SE [28] were used to identify the rRNA and tRNA genes, respectively.

## Genome properties

The genome is 6,703,999 bp and comprised of three replicons (Table 3) with the size of 3,641,423 bp (chromosome), 1,422,736 bp (pSymA) and 1,639,840 bp (pSymB). The average GC content is 61.93%. Three rRNA operons, 67 RNA only genes were identified and 6583 protein coding genes were predicted in the genome. Five thousand thirty-two protein-coding genes were assigned a putative function and 1551 genes were predicted to code for hypothetical proteins (Table 4). The distribution of genes in COG functional categories is presented in Table 5.

**Table 3:** Summary of genome: one chromosome and 2 plasmids

Label	Size (Mb)	Topology	INSDC identifier	RefSeq ID
Chromosome	3.641	Circular	CP019584	NZ_CP019584.1
Plasmid 1	1.640	Circular	CP019586	NZ_CP019586.1
Plasmid 2	1.423	Circular	CP019585	NZ_CP019585.1

**Table 4:** Genome statistics for *E. meliloti* strain FSM-MA

Attribute	Value	% of Total
Genome size (bp)	6,703,999	100.00
chromosome size (bp)	3,641,423	54.32
pSymA size (bp)	1,422,736	21.22
pSymB size (bp)	1,639,840	24.46
DNA coding region (bp)	5,641,977	84.16
DNA G + C content (bp)	4,152,010	61.93
DNA scaffolds	3	100.00
Total genes	6650	100.00
chromosomal genes	3574	53.74
pSymA genes	1481	22.27
pSymB genes	1595	23.98
Protein-coding genes	6183	92.97
RNA genes	67	1.01
Pseudo genes	400	6.01
Genes in internal clusters	2341	35.20
Genes with function prediction	5032	75.67
Genes assigned to COGs	5801	87.23
Genes with Pfam domains	5167	77.70
Genes with signal peptides	534	8.03
Genes with transmembrane helices	1403	21.10
CRISPR repeats	0	0

**Table 5:** Number of genes of *Ensifer meliloti* FSM-MA associated with general COG functional categories

Code	chromosome		pSymA		pSymB		Genome		
	Value	% age of total (3574)	value	% age of total (1481)	value	% age of total (1595)	value	% age of total (6650)	Description
J	164	4.59	7	0.47	16	1.00	187	2.81	Translation, ribosomal structure and biogenesis
A	0	0.00	0	0.00	0	0.00	0	0.00	RNA processing and modification
K	246	6.88	132	8.91	138	8.65	516	7.76	Transcription
L	140	3.92	40	2.70	26	1.63	206	3.10	Replication, recombination and repair
B	1	0.03	0	0.00	0	0.00	1	0.02	Chromatin structure and dynamics
D	30	0.84	5	0.34	10	0.63	45	0.68	Cell cycle control, cell division, chromosome partitioning
V	34	0.95	11	0.74	20	1.25	65	0.98	Defense mechanisms
T	135	3.78	77	5.20	71	4.45	283	4.26	Signal transduction mechanisms
M	148	4.14	32	2.16	104	6.52	284	4.27	Cell wall/membrane/envelope biogenesis
N	55	1.54	12	0.81	6	0.38	73	1.10	Cell motility
U	70	1.96	33	2.23	3	0.19	106	1.59	Intracellular trafficking, secretion, and vesicular transport
O	127	3.55	31	2.09	22	1.38	180	2.71	Posttranslational modification, protein turnover, chaperones
C	177	4.95	121	8.17	75	4.70	373	5.61	Energy production and conversion
G	236	6.60	94	6.35	245	15.36	575	8.65	Carbohydrate transport and metabolism
E	353	9.88	137	9.25	139	8.71	629	9.46	Amino acid transport and metabolism
F	82	2.29	7	0.47	21	1.32	110	1.65	Nucleotide transport and metabolism
H	133	3.72	31	2.09	35	2.19	199	2.99	Coenzyme transport and metabolism
I	117	3.27	37	2.50	53	3.32	207	3.11	Lipid transport and metabolism
P	140	3.92	81	5.47	78	4.89	299	4.50	Inorganic ion transport and metabolism
Q	76	2.13	35	2.36	42	2.63	153	2.30	Secondary metabolites biosynthesis, transport and catabolism
R	399	11.16	172	11.61	169	10.60	740	11.13	General function prediction only
S	361	10.10	87	5.87	121	7.59	569	8.56	Function unknown
W	1	0.03	0	0.00	0	0.00	1	0.02	Extracellular structures
–	349	9.76	299	20.19	201	12.60	849	12.77	Not in COGs

## Insights from the genome sequence

The genome size of FSM-MA falls within the expected size range of 6.65–8.94 Mbp observed in the 33 sequenced 10.1601/nm.1335 genomes that have been deposited in the Integrated Microbial Genomes (IMG) database. The genome of all 10.1601/nm.1335 strains is composed of a circular chromosome and two megaplasmids/chromids, however, certain strains harbour additional replicons too. In strain FSM-MA, however, no additional plasmid was detected. The strain contains three rRNA gene clusters as other 10.1601/nm.1335 strains do. Similarly to other 10.1601/nm.1328 strains, the highest number of genes is assigned to the COG functional categories amino acid transport and metabolism (9.46%), carbohydrate transport and metabolism (8.65%) and transcription (7.76%). An enrichment of the COG functional categories amino acid transport and metabolism, transcription and signal transduction mechanisms is observed in pSymA, while carbohydrate transport and metabolism and cell wall/membrane/envelope biogenesis are overrepresented on pSymB (Table 5).

### Extended insights

Comparing the FSM-MA genome structure with that of other 10.1601/nm.1335 strains using the Mauve software [29] revealed high co-linearity of the chromosomes and the pSymB megaplasmids in contrast to the pSymA plasmids that are highly variable. For example, the average sequence identity between FSM-MA and strain 1021 is 99.4% and their chromosomes and pSymB plasmids are essentially co-linear. The major differences between the chromosomes originated from the insertion of three putative prophages/insertion elements into the FSM-MA genome at genes coding for tRNAs (SMB554__06910: tRNA-Thr, SMB554_09150: tRNA-Lys, SMB554_16265: tRNA-Met). These inserted elements are of approximately 48, 43 and 44 kbp and contains 70, 54 and 34 predicted ORFs, respectively. In the putative prophages at tRNA-Thr and tRNA-Lys, among hypothetical proteins, a number of phage related functions such as terminase, phage portal and capsid proteins (both prophages) as well as ORFs encoding endonucleases, transcriptional regulators, site-specific integrase, DNA ligase, peptidase or peptidoglycan-binding protein are encoded (prophage at tRNA-Lys). The inserted sequence at tRNA-Met seems to contain genes coding for type I restriction-modification system elements, an N_6_-DNA-methylase, chromosome segregation and AAA family ATPases as well as transcriptional regulators among hypothetical proteins. On the other hand, one putative prophage in the 1021 genome at a tRNA-Ser_CGA gene and the *SMc01989-SMc02032* gene cluster coding for transcriptional regulators, membrane transporter and oxido-reductase elements are missing from the FSM-MA genome. The differences between the pSymB plasmids are mainly attributed to mobile genetic elements (IS elements, transposons) that are associated with strain-specific genes, essentially coding for proteins involved in the biosynthesis and transport of strain-specific LPS (lipopolysaccharide) and K-antigen (capsular polysaccharide) surface polysaccharides (discussed later). The pSymA plasmids – that are the carriers of major symbiotic functions such as genes encoding Nod factor biosynthesis and the nitrogenase enzyme and co-factor biosynthesis – have a number of co-linear blocks but have about 80 kbp size difference (FSM-MA > 1021), and more than 200 kbp (>1/7) of the sequences are absent in the other strain.

As the FSM-MA strain is interesting from the symbiotic point of view, we analysed those genes that are important for the development and functioning of the nitrogen-fixing symbioses. The initiation of the symbiotic interaction requires the production of Nod factors with proper chemical structure via the activity of the so-called Nod, Noe and Nol proteins. The FSM-MA genome contains all the known *nod*, *noe* and *nol* genes described in 10.1601/nm.1335. The *nif* and *fix* genes code for the structural elements of the nitrogenase complex (nitrogenase, nitrogenase reductase, electron transport proteins) performing the reduction of atmospheric nitrogen as well as for proteins required for the biosynthesis of co-factors and the assembly of the complex. All these genes – including the ones that are present in multiple copies such as the three fixNOQP operons – can be found in the FSM-MA genome. Notably, despite the high diversity of the 10.1601/nm.1335 pSymA plasmids harbouring these symbiotic genes, the arrangement and the genomic environment of the nodulation and nitrogen fixation genes in FSM-MA and strain 1021 are the same.

Surface polyasaccharides play an essential role during the infection process [4] when bacteria enter the cells of the developing nodules via the infection threads. In the *Medicago* *-* 10.1601/nm.1335 symbiosis, the production of the succinoglycan exopolysaccharide is required for the continuous growth of the infection threads and its lack can be suppressed by the production of galactoglycan or certain capsular polysaccharides. Lipopolysaccharides might also affect both the infection and bacteroid differentiation processes. The organization and genomic environment of genes for the production and transport of the species-specific polysaccahrides EPS I (*exo* and *exs* genes) and EPS II (*exp* genes) as well as of the conserved part (lipidA and O-antigene core) of LPS (chromosomal and pSymB-born genes) and the KPS transporters are the same in the two 10.1601/nm.1335 strains. In contrast, the genes responsible for the production of the strain-specific polysaccharide moieties of LPS and KPS (Additional file 2: Figure S2), located on pSymB, are unique for the given strains.

## Conclusions

The genome sequence of FSM-MA is of particular interest because the strain is highly effective with the most widely used ecotypes, Jemalong and R108 of the model legume *M. truncatula*. Comparative genomics with less and similarly effective strains as well as the creation and use of genomic libraries from FSM-MA has the potential to identify novel symbiotic genes and genes/operons that contribute to the exceptional symbiotic performance of the strain.

## Additional files

Additional file 1: Figure S1. Multilocus Sequence Analysis of 14 genes, *recA*, *gltA*, *glnA*, *ctrA*, *ftsA*, *ftsZ1*, *ftsZ2*, *gyrB*, *dnaK*, *pnp*, *rpoB*, *thrC*, *atpD* and *gap* in *E. meliloti* strains FSM-MA, Sm1021, Su47, Rm41, AK58, AK83, SM11, GR4, BO21CC and BL225C, *E. arboris* strain LMG14919, *E. medicae* strain WSM419 and *E. fredii* strains USDA257, NGR234 and HH103. The concatenated gene sequences (total 23,220 bp) were aligned by ClustalW and a maximum likelihood tree was inferred from the aligned sequences using MEGA ver. 6.0.6 software (Tamura et al., 2007). The tree was estimated using the Tamura-Nei substitution model (Tamura and Nei, 1993). Bootstrap tests were performed with 1000 replications. The inset shows the topology of the maximum likelihood tree. Tamura K, Nei M. (1993). Estimation of the number of nucleotide substitutions in the control region of mitochondrial DNA in humans and chimpanzees. Mol Biol Evol 10: 512–526. Tamura K, Dudley J, Nei M, Kumar S. (2007). MEGA4: molecular evolutionary genetics analysis (MEGA) software version 4.0. Mol Biol Evol 24: 1596–1599. (TIFF 14278 kb)

Additional file 2: Figure S2. Comparison of the organization of genes responsible for the production of the strain-specifc KPS in *E. meliloti* strains FSM-MA, 1021 and Rm41. The gene clusters are located between conserved genes (red arrow) coding for a 3-methyl-2-oxobutanoate-hydroxymethyl transferase (MOBHMT) and a nucleotidyl transferase. Genes determining conserved functions in KPS production such as transport (RkpR, RkpS, RkpS) or chain-length determination (RkpZ) are drawn as solid blue boxes. Open arrows with blue line indicate strain-specific *rkp* genes. Mustard arrows indicate genes conserved between two strains in the region. Open arrows with black line show genes with unknown function or function that could not be related to KPS synthesis. The genes are not drawn to scale. HypProt: hypothetical protein; pAcetylT: putative acetyl transferase; pMethylT: putative methyl transferase; GlycosylT: glycosyl transferase; pLysozime: putative lysozyme; SecCaBProt: putative secreted calcium-binding protein; pMembProt: putative membrane protein. (TIFF 75 kb)

## Abbreviations

Bp: Basepair
EPS: Exopolysaccharide
KPS: Capsular polysaccharide
LPS: lipopolysaccharide
NCR: Nodule-specific cysteine-rich
