# Supplementary figures and images for: The complete genome sequence of Ensifer meliloti strain CCMM B554 (FSM-MA), a highly effective nitrogen-fixing microsymbiont of Medicago truncatula Gaertn
Source: Stand Genomic Sci. 2017 Dec 13;12:75. doi: 10.1186/s40793-017-0298-3 (PMC5729237; doi:10.1186/s40793-017-0298-3)

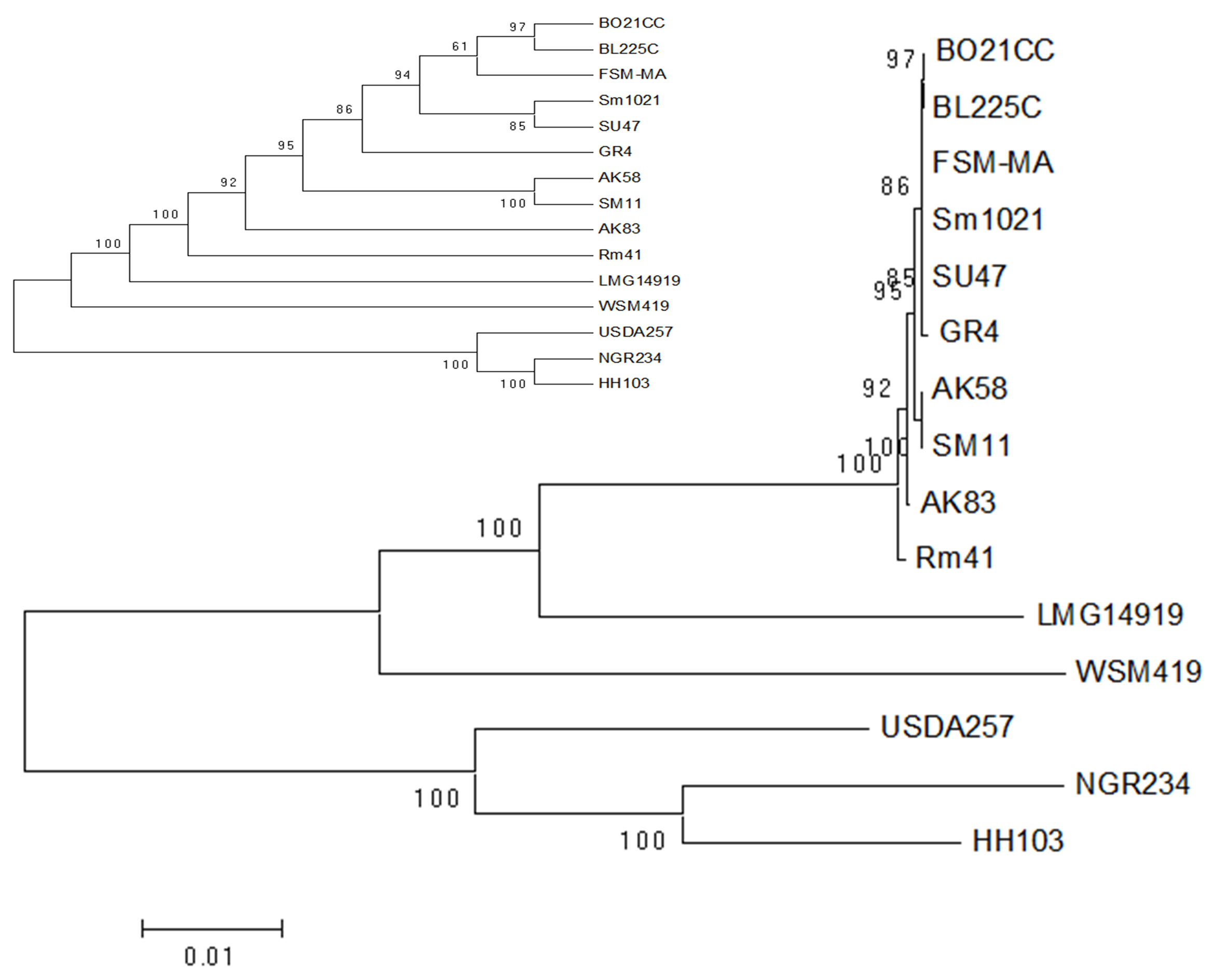

Supplement: Supplementary file 1 — Multilocus Sequence Analysis of 14 genes, recA, gltA, glnA, ctrA, ftsA, ftsZ1, ftsZ2, gyrB, dnaK, pnp, rpoB, thrC, atpD and gap in E. meliloti strains FSM-MA, Sm1021, Su47, Rm41, AK58, AK83, SM11, GR4, BO21CC and BL225C, E. arboris strain LMG14919, E. medicae strain WSM419 and E. fredii strains USDA257, NGR234 and HH103. The concatenated gene sequences (total 23,220 bp) were aligned by ClustalW and a maximum likelihood tree was inferred from the aligned sequences using MEGA ver. 6.0.6 software (Tamura et al., 2007). The tree was estimated using the Tamura-Nei substitution model (Tamura and Nei, 1993). Bootstrap tests were performed with 1000 replications. The inset shows the topology of the maximum likelihood tree. Tamura K, Nei M. (1993). Estimation of the number of nucleotide substitutions in the control region of mitochondrial DNA in humans and chimpanzees. Mol Biol Evol 10: 512–526. Tamura K, Dudley J, Nei M, Kumar S. (2007). MEGA4: molecular evolutionary genetics analysis (MEGA) software version 4.0. Mol Biol Evol 24: 1596–1599. (TIFF 14278 kb) [file 40793_2017_298_MOESM1_ESM.tif]

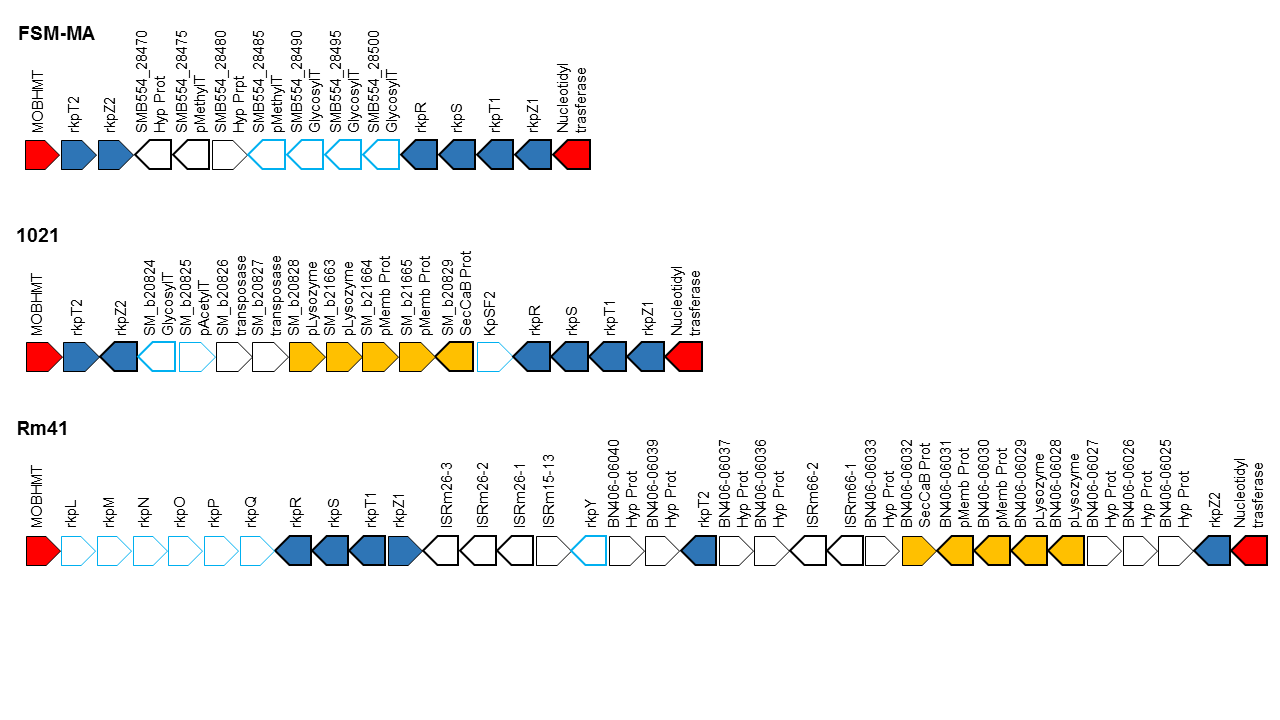

Supplement: Supplementary file 2 — Comparison of the organization of genes responsible for the production of the strain-specifc KPS in E. meliloti strains FSM-MA, 1021 and Rm41. The gene clusters are located between conserved genes (red arrow) coding for a 3-methyl-2-oxobutanoate-hydroxymethyl transferase (MOBHMT) and a nucleotidyl transferase. Genes determining conserved functions in KPS production such as transport (RkpR, RkpS, RkpS) or chain-length determination (RkpZ) are drawn as solid blue boxes. Open arrows with blue line indicate strain-specific rkp genes. Mustard arrows indicate genes conserved between two strains in the region. Open arrows with black line show genes with unknown function or function that could not be related to KPS synthesis. The genes are not drawn to scale. HypProt: hypothetical protein; pAcetylT: putative acetyl transferase; pMethylT: putative methyl transferase; GlycosylT: glycosyl transferase; pLysozime: putative lysozyme; SecCaBProt: putative secreted calcium-binding protein; pMembProt: putative membrane protein. (TIFF 75 kb) [file 40793_2017_298_MOESM2_ESM.tif]
